# Auditory rhythmical cueing to improve gait in community-dwelling stroke survivors (ACTIVATE): a pilot randomised controlled trial

**DOI:** 10.1186/s40814-022-01193-y

**Published:** 2022-11-12

**Authors:** Lisa Shaw, Patricia McCue, Philip Brown, Christopher Buckley, Silvia Del Din, Richard Francis, Heather Hunter, Allen Lambert, Sue Lord, Christopher I. M. Price, Helen Rodgers, Lynn Rochester, Sarah A. Moore

**Affiliations:** 1 Stroke Research Group, Population Health Sciences Institute, Faculty of Medical Sciences, Newcastle University, Newcastle upon Tyne, NE2 4HH UK; 2 Newcastle upon Tyne Hospitals NHS Foundation Trust, Clinical Ageing Research Unit, Campus for Ageing and Vitality, Newcastle upon Tyne, NE4 5PL UK; 3 Department of Sport, Exercise and Rehabilitation, Faculty of Health and Life Science, Northumbria University, Newcastle upon Tyne, NE7 7YT UK; 4 Institute of Translational and Clinical Research, Faculty of Medical Sciences, Newcastle University, Newcastle upon Tyne, NE1 7RU UK; 5 Service user. Contact via Stroke Research Group, Population Health Sciences Institute, Faculty of Medical Sciences, Newcastle University, Newcastle upon Tyne, NE2 4HH UK; 6 Auckland University of Technology, 55 Wellesley St E, Auckland, 1010 New Zealand; 7 Stroke Northumbria, Northumbria Healthcare NHS Foundation Trust, Rake Lane, North Shields, Tyne and Wear, NE29 8NH UK

**Keywords:** Stroke, Gait, Exercise, Rehabilitation, Auditory rhythmical cueing, Pilot randomized controlled trial

## Abstract

**Background:**

Gait impairment limiting mobility and restricting activities is common after stroke. Auditory rhythmical cueing (ARC) uses a metronome beat delivered during exercise to train stepping and early work reports gait improvements. This study aimed to establish the feasibility of a full scale multicentre randomised controlled trial to evaluate an ARC gait and balance training programme for use by stroke survivors in the home and outdoors.

**Methods:**

A parallel-group observer-blind pilot randomised controlled trial was conducted. Adults within 2 years of stroke with a gait-related mobility impairment were recruited from four NHS stroke services and randomised to an ARC gait and balance training programme (intervention) or the training programme without ARC (control). Both programmes consisted of 3x30 min sessions per week for 6 weeks undertaken at home/nearby outdoor community. One session per week was supervised and the remainder self-managed. Gait and balance performance assessments were undertaken at baseline, 6 and 10 weeks. Key trial outcomes included recruitment and retention rates, programme adherence, assessment data completeness and safety.

**Results:**

Between November 2018 and February 2020, 59 participants were randomised (intervention *n*=30, control *n*=29), mean recruitment rate 4/month. At baseline, 6 weeks and 10 weeks, research assessments were conducted for 59/59 (100%), 47/59 (80%) and 42/59 (71%) participants, respectively. Missing assessments were largely due to discontinuation of data collection from mid-March 2020 because of the UK COVID-19 pandemic lockdown. The proportion of participants with complete data for each individual performance assessment ranged from 100% at baseline to 68% at 10 weeks. In the intervention group, 433/540 (80%) total programme exercise sessions were undertaken, in the control group, 390/522 (75%). Falls were reported by five participants in the intervention group, six in the control group. Three serious adverse events occurred, all unrelated to the study.

**Conclusion:**

We believe that a definitive multicentre RCT to evaluate the ARC gait and balance training programme is feasible. Recruitment, programme adherence and safety were all acceptable. Although we consider that the retention rate and assessment data completeness were not sufficient for a future trial, this was largely due to the UK COVID-19 pandemic lockdown.

**Trial registration:**

ISRCTN, ISRCTN10874601, Registered on 05/03/2018,

**Supplementary Information:**

The online version contains supplementary material available at 10.1186/s40814-022-01193-y.

## Key messages regarding feasibility

1. An ARC gait and balance training programme for use by stroke survivors at home and outdoors had been developed, and we wished to establish if evaluation in a full-scale RCT would be possible by reviewing key trial parameters including recruitment and retention rates, programme adherence, gait and balance assessments data completeness and safety.
2. Recruitment rate, programme adherence and safety were all acceptable. Retention rate and assessment data completeness were not sufficient for a future trial, but this was largely due to discontinuation of data collection from mid-March 2020 because of the UK COVID-19 pandemic lockdown.
3. We believe that a definitive multicentre RCT to evaluate the ARC gait and balance training programme is feasible.

## Background

Neurological impairments affecting gait and balance are common features of stroke. Estimates about recovery are variable with studies reporting that between 60 and 80% of survivors can mobilise independently at the end of their hospital stay [1, 2], but only around one quarter regain the ability for real-world walking outside of the home [2, 3]. Improving treatments for gait and balance problems is frequently considered an important research priority for stroke rehabilitation [4, 5] and a very recent UK James Lind research priority setting exercise reconfirmed the need to establish how to deliver therapy to attain best outcomes after stroke as well as facilitate abilities necessary for everyday life [6].

Auditory rhythmical cueing (ARC) uses a metronome beat or music delivered during exercise to train stepping. Although systematic reviews report benefits from using ARC for gait rehabilitation after stroke, including improvements in gait speed, stride length, and cadence [7–9], most studies were conducted in laboratories and therefore have limited applicability to ‘real-world’ situations. Only one small pilot study involving 12 stroke survivors has evaluated an ARC programme within the home. Although this study reported feasibility and improvements in gait parameters [10], one small study alone is insufficient to confirm whether ARC may have a role outside of the laboratory and in wider stroke gait rehabilitation.

We wished to further assess this potentially promising therapy and firstly due to the lack of an available standardised ARC treatment schedule, our team developed an ARC gait and balance training programme for use by stroke survivors in the home and outdoors [11]. Prior to undertaking a full-scale multi-centre randomised controlled trial to evaluate the effects of this new programme, we next considered it important to establish whether conducting such a trial would be feasible. The aim of the study reported here was to assess the suitability of the proposed RCT design by undertaking a pilot trial involving participants from four NHS stroke services.

## Methods

### Objectives

The objectives were to report recruitment and retention, adherence to study treatments, views about the treatments received (i.e., participants) or provided (i.e., staff), data quality and summary statistics from outcome assessments, success of outcome assessor blinding and adverse events.

To determine whether to proceed to a definitive RCT, progression criteria based on published recommendations [12] were set for recruitment rate, treatment adherence and data completeness (Table 1).

**Table 1:** Definitive RCT progression criteria

Criterion	Green	Amber	Red
**Recruitment of participants**	Average of at least four patients per month recruited across the four sites	Average of at least three patients per month recruited across the four sites	Average of two or fewer patients recruited per month across the four sites
**Treatment adherence**	Average of at least 80% supervised and self-monitored treatment sessions completed across the intervention and control groups	Average of at least 70% supervised and self-monitored treatment sessions completed	Average of 70% or less supervised and self-monitored treatment sessions completed
**Data completeness**	Completion (no missing data) of over 85% of key outcome measures at the 10-week outcome assessment	Completion of over 70% of key outcome measures at 10 weeks	Completion of 70% or less of key outcome measures at 10 weeks.

### Study design and setting

The study methods have been reported in detail previously [13]. The design was a parallel-group observer-blind multicentre pilot randomised controlled trial. Participants were recruited from four National Health Service (NHS) stroke services in North East England, UK. Study treatments were delivered in the home and nearby outdoors. Ethical approval was granted by London-City and East Research Ethics Committee (ref 18/LO/0115).

### Participants

NHS stroke staff from the participating services screened patients for potential eligibility and sought permission to pass contact details onto the research team, who confirmed eligibility and obtained consent. Community-dwelling adults within 24 months of stroke with a gait impairment (e.g., gait asymmetry, reduced walking speed, reduced balance; assessed by stroke staff clinical observation and/or patient report) but could mobilise independently indoors (with/without stick) for greater than 10 m were eligible for inclusion. People who had other neurological or orthopedic conditions affecting gait, cardiopulmonary conditions limiting walking, and cognitive/communication issues or a diagnosis likely to interfere with study procedures (e.g., uncorrected hearing problems, registered blind) were excluded. People undergoing active physiotherapy were also excluded. All participants had to be able to provide informed consent.

### Randomisation

A member of the research team used an online randomisation service (https://www.sealedenvelope.com/) to allocate participants to intervention or control in a 1:1 ratio using permuted block sequences. No stratification was used for this pilot trial.

### Blinding

Due to the nature of the interventions, it was not possible to blind participants or treatment providers to study group. However, face-to-face outcome assessments were intended to be conducted by a blinded researcher and any unblinding was recorded.

### Intervention

The ARC gait and balance training programme was developed from a literature review and stakeholder workshops [11]. Consisting of three 30-min sessions per week for 6 weeks (total 18 sessions), one session per week was supervised face-to-face by a trained member of the research team (PM (stroke researcher with background in psychology) or HH (research therapist with over 20 years specialist stroke clinical experience)) and two sessions were self-managed. ARC was provided with either a commercially available metronome (Metro Tuner MT-100 by Musedo) or a free metronome app for a mobile phone (‘ZyMi’ for android or ‘Pro Metronome’ for iOS), according to participant preference. A single tone was used to cue each leg with a regular pattern and the cueing frequency was dependent on exercise type. Ten gait and balance exercises which were gradually progressed according to participant ability were used with ARC. Progression included increasing cueing frequency, increasing number of repetitions or time spent on an exercise and increasing task difficulty such as increasing number of turns. During weeks 4 to 6, the supervised session included outdoor walking. Examples of exercises are shown in Additional files 1 and 2. All participants were provided with a training manual which included illustrations of the exercises and a diary section to record each session undertaken. Videos of the exercises could also be accessed online. The diary section was intended to be easy to complete and requested a tick in a box for each exercise undertaken in each session. An example of the diary is shown in Additional file 3. At the end of the programme, diaries were either collected by the face-to-face outcome assessor or returned to the research team by post. In addition, both participants and staff were asked to complete study-specific feedback forms. A description of the training programme using the Template for Intervention Description and Replication checklist [14] is provided in Additional file 4.

### Control

The gait and balance training programme was undertaken without ARC. The duration, content, supervision, and materials were identical to the programme for participants in the intervention group, excluding the use of ARC.

### Baseline and outcome data collection

Data were collected at baseline (prior to randomisation) and at 6- and 10-weeks post-randomisation. Participant characteristics collected face-to-face by a researcher at baseline included age, sex, pre-stroke disability (modified Rankin Scale [15, 16]), pre-stroke walking status (with/without stick), stroke type, current stroke limitations (impairment: National Institute of Health Stroke Scale [17]; disability: modified Rankin Scale [15, 16]), and cognition (Montreal Cognitive Assessment [18]). Mood (Physical Health Questionnaire-9 [19]) and fatigue (Fatigue Assessment Scale [20]) were recorded in a participant self-completion questionnaire.

At each study time point, ambulatory ability (Functional Ambulation Category (FAC) [21], Rivermead Mobility Index (RMI) [22]), balance/gait (Mini Balance Evaluation Systems Test (Mini-BEST) [23, 24], Activities Specific Balance Confidence (ABC) Scale [25, 26]), and gait speed (4-m walk test) were assessed. These data were collected face-to-face by a researcher except the ABC Scale which was included in a participant self-completion questionnaire.

To assess the safety of the training programmes, adverse events and falls were recorded. Participants were asked to complete a falls diary recording any events which fulfilled a standard fall definition [27]. Training programme providers enquired about falls weekly and assisted with diary completion. At the 6- and 10-week face-to-face assessments, participants were questioned about any new medical problems to capture other adverse events.

Data were also collected on walking activity levels using accelerometer-based wearable sensors (Axivity) and quality of life using questionnaire scales, but these results will be reported separately.

The choice of data to be collected reflected information and measures which we wished to test for feasibility of use in a future RCT. This included stroke characteristics which may influence walking outcome and a range of measures spanning impairment, activity and participation.

### Sample size

In keeping with recommendations for pilot trials, the planned sample size was 60 participants [28].

### Data analysis

As this was a pilot trial, data analyses were descriptive only, group comparisons were not undertaken. Numbers and percentages are reported for categorical variables. Mean and standard deviation (SD) or median and interquartile range (IQR) are reported for continuous variables. For measurement scales, only complete case data are reported, imputation for missing items was not undertaken.

## Results

Between 05 November 2018 and 28 February 2020, 97 patients were recorded as screened for the trial and 60 participants provided consent to take part. The four NHS stroke services commenced their involvement at slightly different times resulting in sites 1 and 2 enrolling participants for 68 weeks, site 3 for 66 weeks and site 4 for 65 weeks. The most common reason recorded for failure to enrol a screened patient was that the patient declined.

Numbers of participants consented per site were site 1: 22 (1.5 patients/month), site 2: 12 (0.8 patient/month), site 3: 10 (0.7 patients/month), and site 4: 16 (1.1 patients/month). Taking the enrolment period as 15 months, a mean of 4 patients per month were consented in total. Of these 60 participants who provided consent, one participant withdrew before randomisation resulting in 59 participants being allocated to a treatment group (intervention group, *n*=30; control group, *n*=29).

**Fig. 1:**
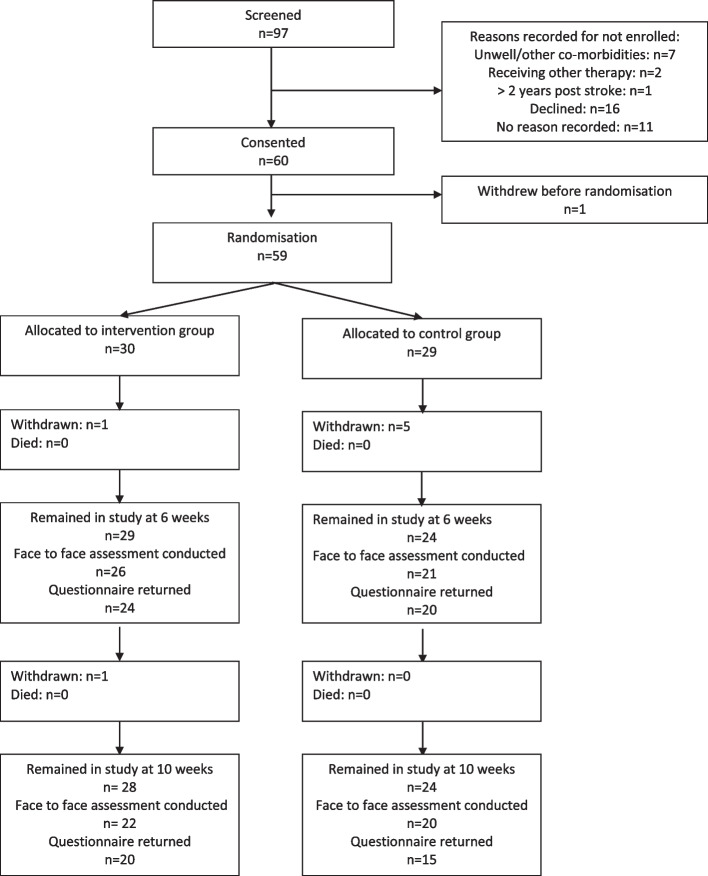
Trial profile

Face-to-face assessments were conducted for 47/59 (80%) of participants at 6 weeks and 42/59 (71%) at 10 weeks. Where the data collection was expected (i.e., the participant had not withdrawn from the study), the only reason for an assessment not being conducted was because the study had to discontinue activity during the UK COVID-19 pandemic lockdown imposed from March 2020. Enrolment had completed prior to the lockdown but the on-going programme training sessions and follow up assessments had to be abandoned. Removing the participants who could not be seen due to the COVID-19 pandemic, face to face assessments were conducted for 47/53 (89%) at 6 weeks and 42/49 (86%) at 10 weeks. Figure 1 shows the trial profile, and Table 2 shows the reasons for missing assessments and questionnaires.

**Table 2:** Reasons for missing study assessments

	Intervention	Control
**Withdrawn at or before a 6-week assessment**	*n*=1 • Ill health: *n*=1	*n*=5 • Ill health: *n*=2 • Unspecified: *n*=3
**Face to face assessment expected but not conducted at 6 weeks**	*n*=3 • COVID-19 restrictions: *n*=3	*n*=3 • COVID-19 restrictions: *n*=3
**Questionnaire expected but not returned**	*n*=5 • COVID-19 restrictions: *n*=4 • Pt did not return: *n*=1	*n*=4 • COVID-19 restrictions: *n*=2 • Pt did not return: *n*=2
**Withdrawn between 6 and 10 weeks**	*n*=1 • Unspecified: *n*=1	*n*=0
**Face to face assessment expected but not conducted at 10 weeks**	*n*=6 • COVID-19 restrictions: *n*=6	*n*=4 • COVID-19 restrictions: *n*=4
**Questionnaire expected but not returned**	*n*=8 • COVID-19 restrictions: *n*=4 • Pt did not return: *n*=4	*n*=9 • COVID-19 restrictions: *n*=4 • Pt did not return: *n*=4 • Not mailed as no response to other questionnaires *n*=1

Baseline characteristics of randomised participants are shown in Tables 3 and 4. The intervention group had slightly more females (33% versus 28%), were marginally older (median 70 years versus 62 years) and randomised into the trial a little later after stroke (9.1 months versus 7.6 months). Results of baseline gait assessments were similar in each group.

**Table 3:** Baseline characteristics

	Intervention	Control
**Sex,** ***n*** **(%)**	*n*=30	*n*=29
Male	20 (67%)	21 (72%)
Female	10 (33%)	8 (28%)
**Age years**	*n*=30	*n*=29
Median [IQR]	70 [65, 78]	62 [58, 77]
Mean (SD)	71 (10)	66 (13)
**Pre-stroke mRS,** ***n*** **(%)**	*n*=30	*n*=29
0	29 (97%)	28 (97%)
1	0 (0%)	0 (0%)
2	0 (0%)	0 (0%)
3	1 (3%)	1 (3%)
4	0 (0%)	0 (0%)
5	0 (0%)	0 (0%)
**Pre-stroke walking status,** ***n*** **(%)**	*n*=30	*n*=29
Stick	5 (17%)	4 (14%)
Without stick	25 (83%)	25 (86%)
**Stroke type,** ***n*** **(%)**	*n*=30	*n*=29
Ischaemic	28 (93%)	26 (90%)
Intracerebral haemorrhage	2 (7%)	3 (10%)
Subarachnoid haemorrhage	0 (0%)	0 (0%)
**Stroke sub-type,** ***n*** **(%)**	*n*=27	*n*=28
TACS	6 (22%)	4 (14%)
PACS	7 (26%)	6 (21%)
LACS	9 (33%)	13 (46%)
POCS	3 (11%)	3 (11%)
Unable to verify	2 (7%)	2 (7%)
**First ever stroke,** ***n*** **(%)**	*n*=30	*n*=29
No	5 (17%)	4 (14%)
Yes	25 (83%)	25 (86%)
***Residual deficit due to previous stroke, n (%)***	*n*=5	*n*=4
*No*	3 (60%)	3 (75%)
*Yes*	2 (40%)	1 (25%)
**Side of body affected by stroke,** ***n*** **(%)**	*n*=30	*n*=29
Right	16 (53%)	11 (38%)
Left	13 (43%)	18 (62%)
Both	1 (3%)	0 (0%)
**Time from stroke to randomisation (days)**	*n*=29	*n*=29
Median [IQR]	273 [219, 390]	229 [155, 307]
Mean (SD)	312 (159)	254 (131)
**Baseline stroke severity (National Institute for Health Stroke Scale) (scored 0 to 42)**	*n*=30	*n*=29
Median [IQR]	3 [2, 4]	4 [3, 6]
Mean (SD)	3 (3)	5 (2)
**Baseline mRS n (%)**	*n*=30	*n*=29
0	0 (0%)	0 (0%)
1	5 (17%)	4 (14%)
2	5 (17%)	6 (21%)
3	20 (67%)	19 (66%)
4	0 (0%)	0 (0%)
5	0 (0%)	0 (0%)
**Baseline cognition (MOCA)** **(scored 0 to 30)**	*n*=30	*n*=29
Median [IQR]	25 [22, 28]	24 [22, 27]
Mean (SD)	24 (5)	24 (4)
**Baseline depression (PHQ-9)** **(scored 0 to 27)**	*n*=25	*n*=27
Median [IQR]	6 [2, 10]	6 [3, 9]
Mean (SD)	6 (5)	6 (5)
**Baseline fatigue (Fatigue Assessment Scale)** **(scored 10 to 50)**	*n*=23	*n*=26
Median [IQR]	21 [18, 26]	22 [18, 26]
Mean (SD)	23 (7)	23 (7)

**Table 4:** Gait and balance performance assessments

	Baseline		Six weeks		10 weeks	
	Intervention	Control	Intervention	Control	Intervention	Control
**Gait speed—4-m walk test** **(time (seconds) to complete 4m)**	*n*=30	*n*=29	*n*=26	*n*=21	*n*=20	*n*=20
Median [IQR]	7.72 [5.73, 10.77]	7.88 5.8 [4, 10.77]	7.17 [5.42, 11.86]	7.39 [4.87, 10.11]	7.97 [5.07, 10.77]	7.35 [5.07, 8.52]
Mean (SD)	10.39 (7.73)	10.00 (6.91)	10.26 (7.50)	8.41 (4.92)	8.84 (4.64)	8.44 (4.83)
**Gait speed—4-m walk test** **(m/s)**	*n*=30	*n*=29	*n*=26	*n*=21	*n*=20	*n*=20
Median [IQR]	0.52 [0.37, 0.70]	0.51 [0.38, 0.69]	0.56 [0.34, 0.74]	0.54 [0.40, 0.83]	0.51 [0.37, 0.79]	0.55 [0.47, 0.79]
Mean (SD)	0.53 (0.23)	0.51 (0.20)	0.54 (0.25)	0.60 (0.25)	0.56 (0.24)	0.59 (0.23)
**Rivermead Mobility Index** (scored 0 to 15)	*n*=30	*n*=29	*n*=26	*n*=21	*n*=22	*n*=20
Median [IQR]	13 [11, 14]	13 [11, 13]	13 [11, 14]	13 [12, 14]	13 [11, 14]	13 [11, 14]
Mean (SD)	12 (3)	12 (3)	12 (2)	12 (2)	12 (2)	12 (2)
**Functional Ambulation Category** (scored 0 to 5)	*n*=30	*n*=29	*n*=26	*n*=21	*n*=21	*n*=20
Min	3	0	3	4	4	4
Max	5	5	5	5	5	5
Median [IQR]	5 [4, 5]	5 [4, 5]	5 [4, 5]	5 [5, 5]	5 [4, 5]	5 [4, 5]
Mean (SD)	5 (1)	4 (1)	5 (1)	5 (<0.5)	5 (1)	5 (1)
**Mini Balance Evaluation Systems Test** (scored 0 to 28)	*n*=29	*n*=27	*n*=25	*n*=21	*n*=20	*n*=20
Median [IQR]	15 [10, 19]	14 [10, 19]	15 [10, 22]	16 [10, 20]	13 [10, 22]	16 [8, 21]
Mean (SD)	15 (6)	14 (5)	15 (6)	15 (5)	15 (6)	15 (7)
**Activities Specific Balance Confidence Scale** (scored 0 to 100)	*n*=23	*n*=26	*n*=23	*n*=18	*n*=19	*n*=15
Median [IQR]	61 [48, 82]	61 [44, 82]	69 [45, 87]	75 [62, 85]	66 [53, 77]	70 [60, 92]
Mean (SD)	61 (20)	61 (23)	68 (21)	75 (14)	65 (19)	70 (19)

Exercise diaries were available from 28/30 (93%) participants in the intervention group and 25/29 (86%) participants in the control group. For the intervention group, both missing diaries related to participants who did not complete any of the programme (withdrawal *n*=1, suspension of study activity due to the COVID-19 pandemic *n*=1). In the control group, for 1 participant a missing diary was due to withdrawal before completion of any of the programme but for 3 participants, trial notes indicate some involvement in the programme and it was unclear why the diaries were missing.

As there were 18 training sessions in the study programmes, the total number of sessions expected to be undertaken was 540 for 30 intervention group participants and 522 for 29 control group participants. Total recorded sessions in the available dairies were 433/540 (80%) for the intervention group and 390/522 (75%) for the control group. Considering only the participants who returned a diary, the expected sessions drop to 504 in the intervention group and 450 in the control group, giving recorded sessions as 433/504 (86%) and 390/450 (87%), respectively; the median number of sessions recorded per participant was intervention group 16, control group 18.

Participant and training programme provider responses to the study-specific programme feedback questions are shown in Additional files 5 and 6. The majority of participants in both groups reported finding the programme easy to follow and would recommend to others. The providers reported that the duration and content of the programme were appropriate for most participants and materials were acceptable.

Summary statistics for gait and balance performance assessments are shown in Table 4. For both the RMI and the FAC, most participants obtained maximal or near maximal scores across all assessment time points indicating ceiling effects. For other assessments, there was more scope for potential change. Anecdotal feedback from assessors about the use of the Mini-BEST assessment expressed potential safety concerns related to items assessing reactive postural control.

To assess trial data completeness, the proportion of patients contributing data for each gait performance assessment (i.e., including where assessments were not undertaken), was calculated and is shown in Table 5. Over both study groups, data completeness for the assessments collected face-to-face ranged from 100% (most scales at baseline) down to 67.8% (some scales at week 10). For the self-completion questionnaire, data completeness was lower at each time point. Note that these calculations include participants who could not be assessed due to the COVID-19 pandemic.

**Table 5:** Availability of gait and balance performance assessment data

	Baseline data availability			6 week data availability			10 week data availability		
	Intervention	Control	Overall	Intervention	Control	Overall	Intervention	Control	Overall
**Gait speed**	30/30 (100%)	29/29 (100%)	59/59 (100%)	26/30 (86.7%)	21/29 (72.4%)	47/59 (79.7%)	20/30 (66.6%)	20/29 (69.9%)	40/59 (67.8%)
**Rivermead Mobility Index**	30/30 (100%)	29/29 (100%)	59/59 (100%)	26/30 (86.7%)	21/29 (72.4%)	47/59 (79.7%)	22/30 (73.3%)	20/29 (69.9%)	42/59 (71.2%)
**Functional Ambulatory category**	30/30 (100%)	29/29 (100%)	59/59 (100%)	26/30 (86.7%)	21/29 (72.4%)	47/59 (79.7%)	21/30 (70.0%)	20/29 (69.9%)	41/59 (69.5%)
**Mini Balance Evaluation Systems Test**	29/30 (96.7%)	27/29 (93.1%)	56/59 (94.9%)	25/30 (83.3%)	21/29 (72.4%)	46/59 (78.0%)	20/30 (66.6%)	20/29 (69.9%)	40/59 (67.8%)
**Activities Specific Balance Confidence**	23/30 (76.6%)	26/29 (89.7%)	49/59 (83.1%)	23/30 (76.7%)	18/29 (62.1%)	41/59 (69.5%)	19/30 (63.3%)	15/29 (51.7%)	34/59 (57.6%)

At 6 weeks, the assessor reported that they were unblinded for 6/26 (23%) participants in the intervention group and 2/21 (10%) in the control group. At 10 weeks, the figures were 4/22 (18%) and 1/18 (6%), respectively. The main reasons for unblinding were reported to be information provided by the participant or because the metronome was clearly visible.

Three serious adverse events (SAE) were reported during the study, considered to be SAEs due to hospitalisation (urinary catheter issue, seizure, possible new stroke). None were considered related to the study programmes and all occurred in control group participants.

Falls diaries were available for 27/30 (90%) of the intervention group and 24/29 (83%) control group. For 2/3 missing in the intervention group, trial notes indicate that these participants did not take part in any of the programme. For 1/3, it is unclear why the diary was missing. For the control group, for 1/5 missing, trial notes indicate no participation in the programme, and for the remaining 4/5, it is unclear why the diary was missing. Considering the available diaries, in the intervention group 4/27 (15%) participants had a fall recorded and for the control group this was 5/24 (21%). In the control group, one participant had 2 falls recorded. The free text provided described minor injuries only although one incident resulted in attendance of an ambulance but without conveyance to hospital. Other trial notes indicate that two additional participants (intervention *n*=1, control *n*=1) had a fall but these were not registered in diaries.

## Discussion

This pilot trial aimed to establish the feasibility of undertaking a full scale multi-centre RCT to evaluate the ARC gait and balance training programme. Progression criteria based on published recommendations [12] were pre-set for recruitment rate, treatment adherence and data completeness (Table 1). Results obtained suggest that progression to a definitive multicentre RCT is feasible. A number of design and/or delivery aspects can however be strengthened to improve the chances of completing a high-quality evaluation.

Recruitment was an average of 4 participants per month which makes the recruitment progression criterion ‘green’. However, two of the four NHS stroke services did not manage to enrol one participant every month. Whilst recruitment differences are unsurprising, it highlights the need for careful planning of realistic service recruitment rates and/or careful selection of sites likely to fulfil expectations. Although aiming for recruitment of up to one participant per site per month may seem low, this is not unusual for stroke rehabilitation trials [29, 30] and would not make a future trial prohibitive. In planning recruitment targets for stroke rehabilitation studies, delivery of the rehabilitation intervention must also be considered as this typically requires face-to-face staff time and as such limits the number of people who can receive a study treatment at any time.

Although we collected screening data, unfortunately, these data do not help to explain the different site recruitment rates. In hindsight, the detail captured by the screening log was limited as only one reason for failure to enrol was requested rather than firstly asking staff to record whether or not a patient met the eligibility criteria, and secondly whether or not they were willing to take part. Had these factors been separately recorded as part of the screening log, it would have been possible to report whether a specific eligibility criterion was limiting enrolment independently to whether or not patients were willing to take part. This would have allowed eligibility to be reviewed and potentially updated before a larger study. In addition, the screening data were likely incomplete as logs recorded only 97 screened patients and many more people with post-stroke gait impairment would have been managed by the four participating NHS services over the study 65–68 week timeframe. For a future trial, the screening log should be improved to capture appropriate eligibility information which could be used to inform live recruitment rates, and completion should be encouraged. However, although this would be a recommendation, our experience of leading other trials suggests that screening data is generally considered a low priority by participating sites and this would be difficult to implement.

Adherence to the study training programmes was assessed by counting the number of sessions that participants and/or providers recorded as undertaken. Considering just the participants who returned a diary, the adherence progression criterion was ‘green’ with over 80% of sessions recorded as undertaken in both study groups. Furthermore, as this included some participants who had to discontinue participation due to the UK COVID-19 lockdown, it is likely that adherence would have been higher if this disruption had not occurred. However, if all enrolled participants are included in calculations, adherence is ‘amber’ at 80% in the intervention group and 75% in the control group. This difference occurs due to participants with missing diaries being considered to have completed 0 sessions, but this may be an underestimate as trial notes indicate that some people with missing diaries engaged with the programme.

Assessment of adherence by counting sessions alone is limited as this does not provide information on whether the intended 30 min of exercises was undertaken nor whether the content was as planned. However, as 12/18 sessions in the programmes were self-managed, asking participants to complete more complex information may have affected the good diary return rates observed. Nevertheless, consideration should be given to whether richer programme adherence data could be captured in a future large trial.

The data completeness progression criterion was ‘red,’ but this was affected by the inability to collect data because of the COVID-19 pandemic lockdown. Prior to this, data collection was proceeding well and this progression criterion would likely have been ‘green’ without the lockdown. Unsurprisingly, participant self-completion questionnaire data were less complete than face-to-face collected data and the use of self-completion material should be reconsidered for a main trial.

Summary statistics for the RMI and FAC indicated ceiling effects in our study population and as such would unlikely be suitable choices for outcome assessments in a future RCT. Due to the safety concerns about Mini-BEST assessment, we also consider this measure unsuitable for further use. Gait speed and the ABC scale would be the most appropriate performance measures to be retained for a larger trial.

Face-to-face assessment blinding to treatment group was not achieved with all participants which is a recognised problem in rehabilitation trials where masking receipt of the study treatments is not possible [31, 32].

Monitoring of safety was an important aspect of this pilot trial and there were no serious adverse events related to the training programmes. Records indicated that 11 participants sustained a fall during the study timeframe with details available for 9/11 showing minor injuries only. Fall diaries were missing for some participants and therefore our data may potentially underestimate falls. In addition, although study staff regularly checked falls diaries and encouraged completion, like any self-completion documentation, there could still be omissions. However, there was no evidence that falls were a cause for concern in the study.

A limitation of this pilot trial is that it was conducted in NE England only and programme supervision was undertaken by researchers. In a future full-scale trial, other regions would be included and routine NHS staff supervision of the programme would be preferable for real-world evidence generation. In addition, for this pilot study, we chose to provide control group participants with the gait and balance training programme but without ARC, as this gave an opportunity for additional data collection about this content; however, for a future definitive RCT, the design should include a group which receives standard care.

## Conclusions

We believe that a definitive multicentre RCT to evaluate the ARC gait and balance training programme is feasible. Recruitment, programme adherence and safety were all acceptable. Although the retention rate and assessment data completeness were not sufficient for a future trial, this was largely due to the UK COVID-19 pandemic lockdown.

## Supplementary Information

**Additional file 1: Figure S1.** Example gait training exercise.

**Additional file 2: Figure S2.** Example balance training exercise.

**Additional file 3: Figure S3.** Example of the diary used to record training sessions.

**Additional file 4: Table S1.** Description of the ARC gait and balance training programme using TIDieR checklist.

**Additional file 5: Table S2.** Participant feedback data.

**Additional file 6: Table S3.** Training programme provider feedback data.

## Data Availability

Data analysed for this study are available from the corresponding author on reasonable request.

## Abbreviations

ARC: Auditory rhythmical cueing
IQR: Interquartile range
NHS: National Health Service
RCT: Randomised controlled trial
SAE: Serious adverse event
SD: Standard deviation
TIDieR: Template for intervention description and replication
