# Supplementary material for: Auditory rhythmical cueing to improve gait in community-dwelling stroke survivors (ACTIVATE): a pilot randomised controlled trial
Source: Pilot Feasibility Stud. 2022 Nov 12;8:239. doi: 10.1186/s40814-022-01193-y (PMC9652598; doi:10.1186/s40814-022-01193-y)
Supplement: Supplementary file 1 — Additional file 1: Figure S1. Example gait training exercise. [file 40814_2022_1193_MOESM1_ESM.docx]

**Auditory rhythmical cueing to improve gait in community dwelling stroke survivors (ACTIVATE): a pilot randomised controlled trial: Additional file**

**Figure 1: Example gait training exercise**

**Exercise: Manoeuvring Between Objects**

**What you will need to do:**

1. Stand up tall and try not to look at your feet.
2. Start the metronome and listen for the beat.
3. Walk to the beat to the obstacles.
4. Manoeuvre around the obstacles keeping in time to the beat.
5. To help with your balance you may touch the backs of the chairs as you turn.

**A B**


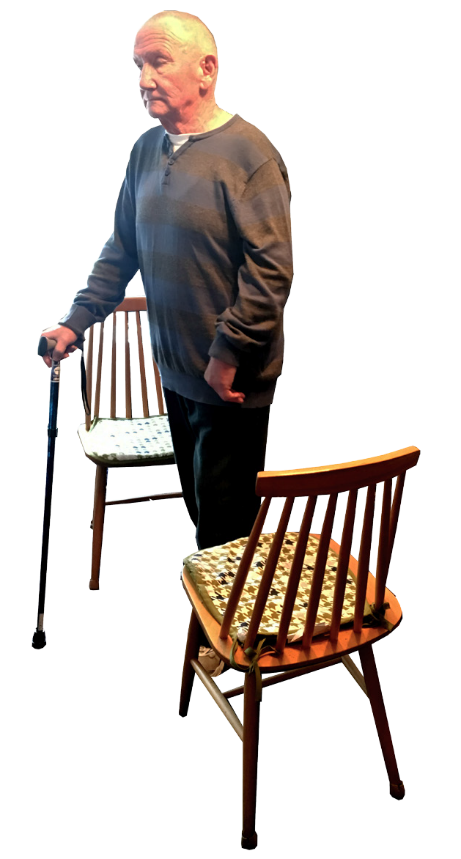

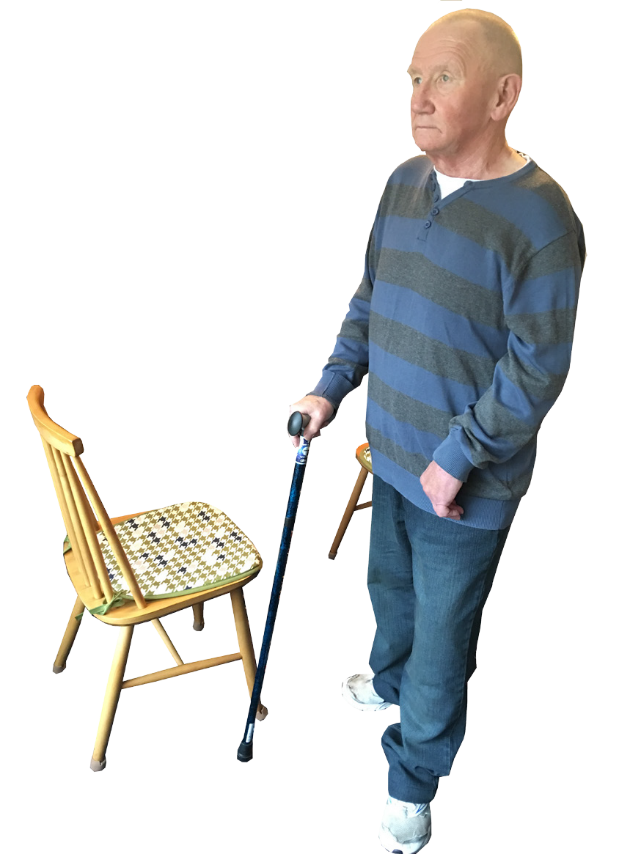


**Setting:** Walking around obstacles in the home e.g. 2 chairs or objects on the floor so the walk can be done in “a figure of eight”

**Repetitions – 1^st^ set:** Repeat each circuit 5 times (e.g. manoeuvring between two chairs

| Progression 1 | Progression 2 | Progression 3 |
| --- | --- | --- |
| Increase the number of circuits | Increase the cueing frequency by 5% | Reduce the cueing frequency by 5% to work on control |
