# Supplementary material for: Auditory rhythmical cueing to improve gait in community-dwelling stroke survivors (ACTIVATE): a pilot randomised controlled trial
Source: Pilot Feasibility Stud. 2022 Nov 12;8:239. doi: 10.1186/s40814-022-01193-y (PMC9652598; doi:10.1186/s40814-022-01193-y)
Supplement: Supplementary file 2 — Additional file 2: Figure S2. Example balance training exercise. [file 40814_2022_1193_MOESM2_ESM.docx]

**Figure 2: Example balance training exercise**

**Exercise: Stepping Forwards and Backwards**

**What you will need to do:**

1. Stand up tall with your feet hip-width apart holding onto a surface for support to one side (e.g. kitchen bench).
2. Start the metronome and listen for the beat.
3. Step your left leg forward.
4. Step your right leg forward so it is parallel with your left leg.
5. Step your left leg back, then your right leg to the starting position.
6. Keep standing up tall.


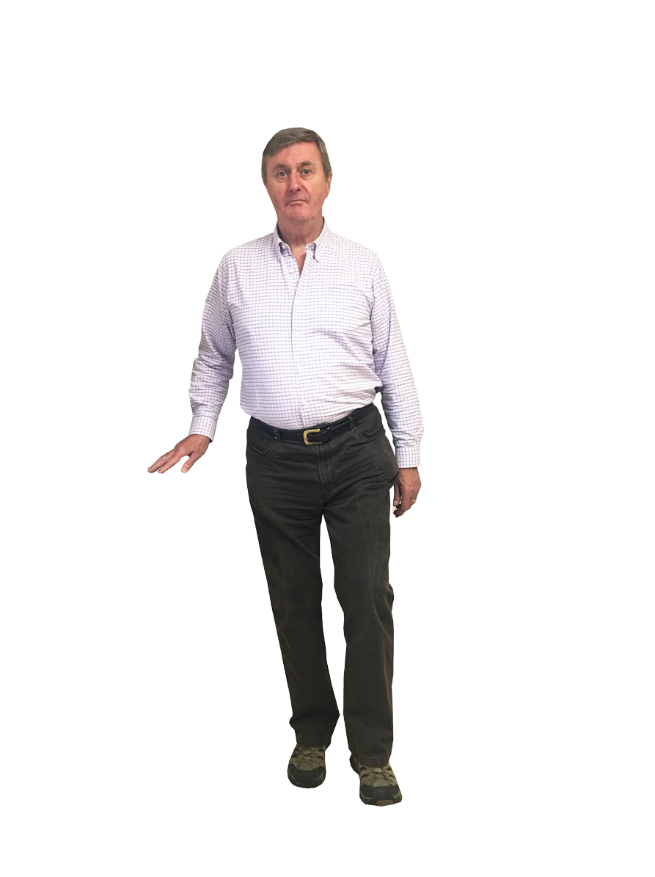

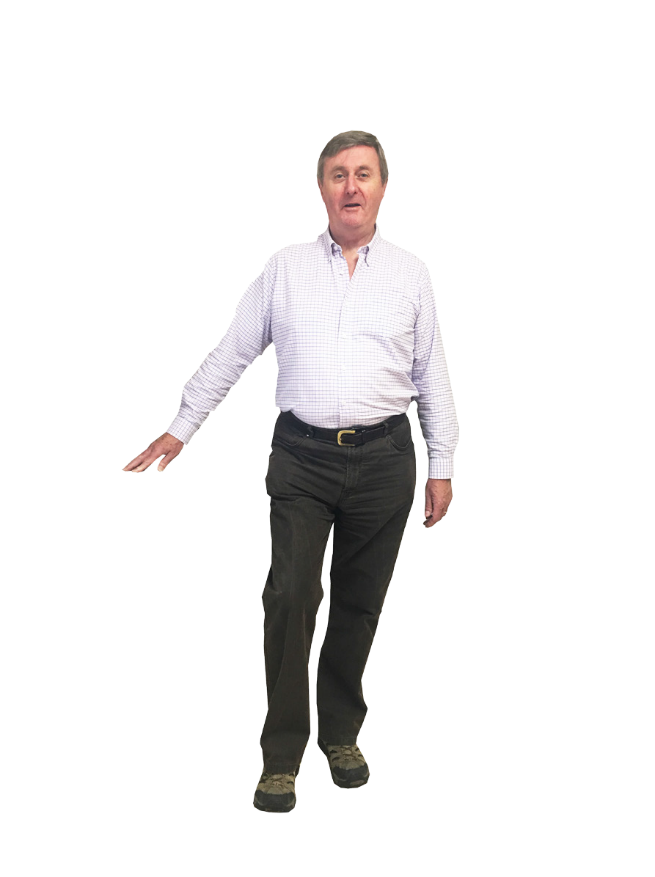


1. Keep your bottom tucked under.

**A B**

**Setting:** Kitchen side/standing by a chair/table

**Repetitions – 1^st^ set:** Repeat 10 times with the left leg then 10 times with the right leg

| Progression 1 | Progression 2 | Progression 3 |
| --- | --- | --- |
| Don’t hold on | Increase the cueing frequency by 5% | Reduce the cueing frequency by 5% to work on control |
