# Supplementary figures and images for: Auditory rhythmical cueing to improve gait in community-dwelling stroke survivors (ACTIVATE): a pilot randomised controlled trial
Source: Pilot Feasibility Stud. 2022 Nov 12;8:239. doi: 10.1186/s40814-022-01193-y (PMC9652598; doi:10.1186/s40814-022-01193-y)

**Figure 3: Example of the diary used to record training sessions**

**
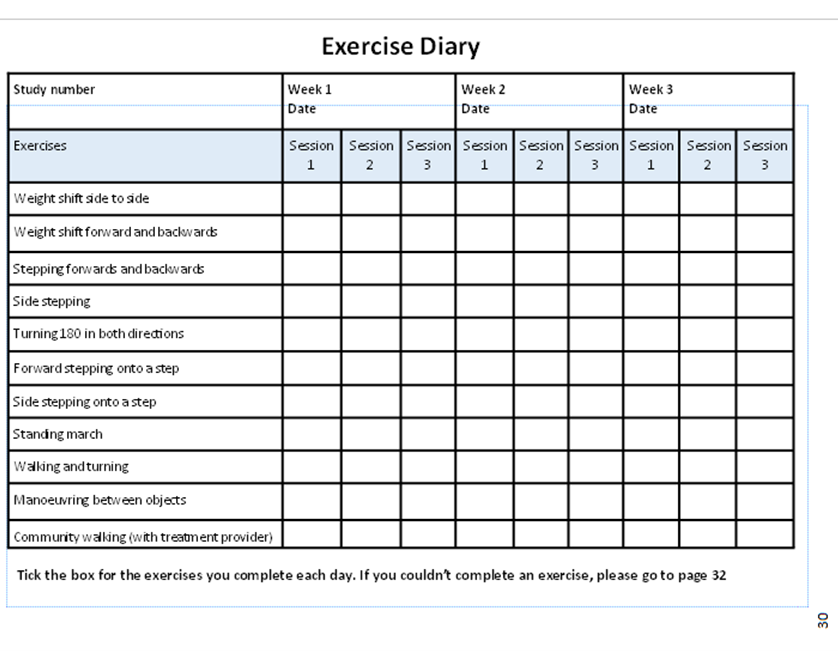
**

Supplement: Supplementary file 3 — Additional file 3: Figure S3. Example of the diary used to record training sessions. [file 40814_2022_1193_MOESM3_ESM.docx]
