# Supplementary material for: Auditory rhythmical cueing to improve gait in community-dwelling stroke survivors (ACTIVATE): a pilot randomised controlled trial
Source: Pilot Feasibility Stud. 2022 Nov 12;8:239. doi: 10.1186/s40814-022-01193-y (PMC9652598; doi:10.1186/s40814-022-01193-y)
Supplement: Supplementary file 4 — Additional file 4: Table S1. Description of the ARC gait and balance training programme using TIDieR checklist. [file 40814_2022_1193_MOESM4_ESM.docx]

**Table 1: Description of the ARC gait and balance training programme using TIDieR checklist**

| TIDieR component | Description |
| --- | --- |
| What (materials): | - Participant manual which included pictures and descriptions of the exercises to be completed - Access to exercise videos online: [https://youtu.be/INlddw1TugA](about:blank) - Metronome: Musedo Metro Tuner MT-100 - Metronome app: ’ZyMi’ for android or ‘Pro Metronome’ for iOS. |
| What (procedures) | A total of 10 different gait and balance exercises undertaken with auditory rhythmical cueing. Exercises were carried out in the home and outdoors. |
| Who provided | A research physiotherapist with specialist stroke skills and over 20 years clinical experience (HH), and a stroke researcher with a background in psychology (PM). |
| How (delivery) | The programme consisted of 18 sessions over six weeks. Six were face-to-face sessions with the providers described above (once per week) and 12 were self-managed sessions (two per week). Telephone support was available if required. All outdoor walking sessions were face-to-face. |
| Where | Participants’ homes and nearby outdoors. |
| When and how much | Eighteen 30 minute sessions, at three per week for six weeks. One session per week was face-to-face with a provider and two sessions were self-managed. |
| Tailoring | Exercises were gradually progressed according to patient ability including by increasing the speed, number of repetitions, task difficulty . Outdoor walking was introduced at week 4. |
