# Supplementary material for: Auditory rhythmical cueing to improve gait in community-dwelling stroke survivors (ACTIVATE): a pilot randomised controlled trial
Source: Pilot Feasibility Stud. 2022 Nov 12;8:239. doi: 10.1186/s40814-022-01193-y (PMC9652598; doi:10.1186/s40814-022-01193-y)
Supplement: Supplementary file 5 — Additional file 5: Table S2. Participant feedback data. [file 40814_2022_1193_MOESM5_ESM.docx]

**Table 2: Participant feedback data**

|  | Intervention | Control |
| --- | --- | --- |
| **I found it easy to participate in the ACTIVATE study n (%)**  Strongly disagree  Disagree  Undecided  Agree  Strongly agree | n=20  0 (0%)  0 (0%)  0 (0%)  6 (30%)  14 (70%) | n=17  0 (0%)  1 (6%)  0 (0%)  9 (53%)  7 (41%) |
| **I found the exercise sheets/videos easy to follow during the unsupervised sessions n (%)**  Strongly disagree  Disagree  Undecided  Agree  Strongly agree | n=20  0 (0%)  1 (5%)  0 (0%)  10 (50%)  9 (45%) | n=17  0 (0%)  0 (0%)  2 (12%)  6 (35%)  9 (53%) |
| **I found it easy to do the exercises to the beat of the metronome n (%)**  Strongly disagree  Disagree  Undecided  Agree  Strongly agree | n=20  0 (0%)  0 (0%)  0 (0%)  13 (65%)  7 (35%) | n/a |
| **I had enough information to do the exercises without the therapist n (%)**  Strongly disagree  Disagree  Undecided  Agree  Strongly agree | n=20  1 (5%)  0 (0%)  0 (0%)  11 (55%)  8 (40%) | n=17  1 (6%)  0 (0%)  1 (6%)  10 (59%)  5 (29%) |
| **It was helpful in improving the way that I walk n (%)**  Strongly disagree  Disagree  Undecided  Agree  Strongly agree | n=20  0 (0%)  0 (0%)  6 (30%)  5 (25%)  9 (45%) | n=17  0 (0%)  0 (0%)  3 (18%)  9 (53%)  5 (29%) |
| **It built confidence in overcoming barriers related to walking n (%)**  Strongly disagree  Disagree  Undecided  Agree  Strongly agree | n=20  0 (0%)  0 (0%)  5 (25%)  7 (35%)  8 (40%) | n=17  0 (0%)  0 (0%)  5 (29%)  8 (47%)  4 (24%) |
| **I felt safe doing the exercise programme n (%)**  Strongly disagree  Disagree  Undecided  Agree  Strongly agree | n=20  0 (0%)  0 (0%)  0 (0%)  8 (40%)  12 (60%) | n=16  0 (0%)  0 (0%)  0 (0%)  9 (56%)  7 (44%) |
| **I would recommend the exercise programme to other people who have problems with walking after stroke n (%)**  Strongly disagree  Disagree  Undecided  Agree  Strongly agree | n=19  0 (0%)  0 (0%)  2 (11%)  2 (11%)  15 (79%) | n=16  0 (0%)  0 (0%)  0 (0%)  5 (31%)  11 (69%) |
