# Supplementary material for: Auditory rhythmical cueing to improve gait in community-dwelling stroke survivors (ACTIVATE): a pilot randomised controlled trial
Source: Pilot Feasibility Stud. 2022 Nov 12;8:239. doi: 10.1186/s40814-022-01193-y (PMC9652598; doi:10.1186/s40814-022-01193-y)
Supplement: Supplementary file 6 — Additional file 6: Table S3. Training programme provider feedback data. [file 40814_2022_1193_MOESM6_ESM.docx]

**Table 3: Training programme provider feedback data**

|  | Intervention | Control |
| --- | --- | --- |
| **The length of the face to face sessions of 30 minutes was adequate to teach the protocol n (%)**  Strongly disagree  Disagree  Undecided  Agree  Strongly agree | n=15  0 (0%)  0 (0%)  1 (7%)  8 (53%)  6 (40%) | n=15  0 (0%)  0 (0%)  0 (0%)  5 (33%)  10 (67%) |
| **The 18 x 30 minute sessions were an appropriate length for the participant to target their gait and balance n (%)**  Strongly disagree  Disagree  Undecided  Agree  Strongly agree | n=15  0 (0%)  0 (0%)  2 (13%)  8 (53%)  5 (33%) | n=15  0 (0%)  0 (0%)  0 (0%)  6 (40%)  9 (60%) |
| **The intervention exercises and progressions were appropriate for the participant n (%)**  Strongly disagree  Disagree  Undecided  Agree  Strongly agree | n=15  0 (0%)  0 (0%)  1 (7%)  8 (53%)  6 (40%) | n=15  0 (0%)  0 (0%)  1 (7%)  3 (20%)  11 (73%) |
| **I found the handbook and falls diary useful for delivering the intervention n (%)**  Strongly disagree  Disagree  Undecided  Agree  Strongly agree | n=15  0 (0%)  0 (0%)  2 (13%)  6 (40%)  7 (47%) | n=15  0 (0%)  0 (0%)  0 (0%)  4 (27%)  11 (73%) |
| **I feel that the videos were a useful resource for delivering the intervention n(%)**  Strongly disagree  Disagree  Undecided  Agree  Strongly agree  *For those reporting ‘strongly disagree’ free text comments stated that videos were not used.* | n=15  3 (20%)  0 (0%)  12 (80%)  0 (0%)  0 (0%) | n=15  1 (7%)  0 (0%)  13 (87%)  1 (7%)  0 (0%) |
| **I feel that the combination of face to face and self-managed sessions were adequate to administer the intervention properly n (%)**  Strongly disagree  Disagree  Undecided  Agree  Strongly agree | n=15  0 (0%)  1 (7%)  1 (7%)  5 (33%)  8 (53%) | n=15  0 (0%)  0 (0%)  0 (0%)  4 (27%)  11 (73%) |
| **I feel that the telephone support sessions format was adequate for the needs of the participant n (%)**  Strongly disagree  Disagree  Undecided  Agree  Strongly agree  *For those reporting ‘strongly disagree’ free text comments stated that telephone support was not used.* | n=15  2 (13%)  0 (0%)  4 (27%)  4 (27%)  5 (33%) | n=15  1 (7%)  0 (0%)  3 (20%)  4 (27%)  7 (47%) |
| **The home setting of the sessions was appropriate for delivery of the intervention n (%)**  Strongly disagree  Disagree  Undecided  Agree  Strongly agree | n=15  0 (0%)  0 (0%)  0 (0%)  4 (27%)  11 (73%) | n=15  0 (0%)  0 (0%)  0 (0%)  5 (33%)  10 (67%) |
